# Development of Novel Thermal Diffusivity Analysis by Spot Periodic Heating and Infrared Radiation Thermometer Method

**DOI:** 10.3390/ma13214848

**Published:** 2020-10-29

**Authors:** Sho Nagata, Tsuyoshi Nishi, Shugo Miyake, Naoyoshi Azuma, Kimihito Hatori, Takaaki Awano, Hiromichi Ohta

**Affiliations:** 1 Graduate School of Science and Engineering, College of Engineering, Ibaraki University, Hitachi, Ibaraki 316-8511, Japan; 19nm939t@vc.ibaraki.ac.jp (S.N.); tsuyoshi.nishi.75@vc.ibaraki.ac.jp (T.N.); 18nd111x@vc.ibaraki.ac.jp (N.A.); 2 Department of Mechanical Engineering, Kobe City College of Technology, Kobe, Hyogo 651-2194, Japan; miyake@kobe-kosen.ac.jp; 3 Hudson Laboratory, Bethel Corporation, Tsuchiura, Ibaraki 300-0037, Japan; k-hatori@btl-hrd.jp (K.H.); t-awano@btl-hrd.jp (T.A.)

**Keywords:** thermal diffusivity analysis, spot periodic heating and infrared radiation thermometer method, the distance-variation method, accuracy of thermal diffusivity, 65.40.-b (Thermal properties of crystalline solids)

## Abstract

A spot periodic heating method is a highly accurate, non-contact method for the evaluation of anisotropy and relative thermophysical property distribution. However, accurately evaluating thermal diffusivity is difficult due to the influence of temperature wave reflection from the whole surface of the sample. This study proposes a method to derive thermal diffusivity using a parameter table based on heat transfer equations using the concept of optimum distance between heating-point and measurement point. This method considers finite sample size, sensitivity distribution of infrared ray detector, intensity distribution of heating laser and sample thickness. In these results, the obtained thermal diffusivity of pure copper corresponded well with previous literature values. In conclusion, this method is considered highly effective in evaluating the thermal diffusivity in the horizontal direction.

## 1. Introduction

As electronic devices continue to improve with multi-functions, increasing stress is being placed on integrated electronic components. Hence, these electronic devices are prone to malfunctions such as thermal runaway and disconnection [1,2,3,4,5,6]. To prevent these problems, it is necessary to perform precise thermal design by numerical calculation, which requires accurate values of thermophysical properties. A spot periodic heating and infrared radiation thermometer method is a widely used technique for measuring the thermal conductivities of functional materials with special shapes, such as sheets, films, and fibers [7,8,9,10,11,12,13,14]. This method is implemented by a laser heating system to create a periodic heat spot on the surface of a sample. This instrument can provide two independent methods: frequency-variation with a fixed distance and distance-variation with a fixed frequency. The former is the method for the evaluation of the thermal diffusivity in the vertical direction of the sample, while the second is a method for the evaluation of the thermal diffusivity in the horizontal direction of the sample [15,16].

**Figure 1:**
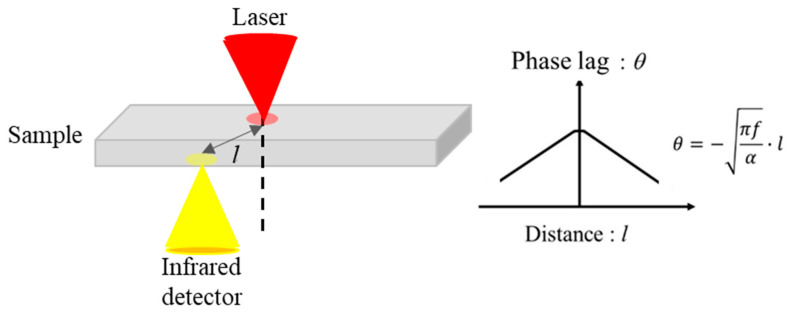
Schematic of the thermowave analyzer (TA) based on the spot periodic heating and infrared radiation thermometer method.

A schematic of the thermowave analyzer (TA) based on the spot periodic heating and infrared radiation thermometer method is shown in Figure 1.

If there is a periodic point heat source that liberates heat at a rate of $P_{0}e^{i\omega t}$ inside an isotropic infinite medium, the thermal diffusivity and specific heat capacity of which is α and c per unit volume, respectively, it generates a temperature wave $T_{ac}$ described by the following equation [17];

(1) $$T_{ac}=\frac{P_{0}}{4\pi \alpha lc}e^{-kt+i\left(\omega t-kl\right)}$$

where l is the distance from the point heat source and k is the wavenumber defined as

(2) $$k=\sqrt{\frac{\omega }{2\alpha }}=\sqrt{\frac{\pi f}{\alpha }}=\frac{1}{\mu }$$

where f is the frequency. The thermal diffusion length μ, which is also defined as in Equation (2), is the reciprocal of k. Therefore, the phase lag θ, which is the difference between phases of surface periodic heating and detected infrared radiation intensity, is expressed as

(3) $$\theta =-\sqrt{\frac{\pi f}{\alpha }}\;l$$

In the case of the distance-variation method, the phase lag is proportional to the distance. Since the frequency was fixed, the in-plane thermal diffusivity can be obtained by the gradient of Equation (3) (Figure 1). Detailed information regarding the thermal diffusivity analysis can be found in a previous study [3]. In actual measurement, the relationship between the heating-detection point distance *l* and the phase lag *θ* in Equation (3) is non-linear. We considered the reason why the non-linearity occurs as follows:

1. The distribution of the intensity of the heating laser beam and the intensity of the infrared detector cannot be ignored.
2. The effect of the reflection of temperature waves from the upper and lower surface cannot be ignored.

Therefore, it is necessary to analyze the region where the influence of temperature wave reflection is not large. In this study, a heat transfer parameter table standardized from the thermal diffusivity and heating frequency was created by the calculation considering the finiteness of the sample thickness and temperature measurement system using an infrared detector, and the obtained table. The optimum heating-detection distance was determined for each sample thickness, thermal diffusivity, and heating frequency were used for measurement. Thus, it becomes possible to analyze thermal diffusivity in the accurate analysis region. A measurement experiment using pure copper was performed in this study.

## 2. Materials and Methods

When calculating the thermal diffusivity in the periodic heating method, the obtained phase lag is affected by the reflection of the temperature wave from the upper and lower surface of the sample. This study suggests a method to obtain the optimum heating-detection distance for each sample thickness and each thermal diffusion length by simulation. First, the Gaussian distribution of the laser and the spatial sensitivity distribution of the detector are evaluated. The phase lag was calculated by using the heating-detection distance where the influence of the reflection of the temperature wave on each sample thickness and each thermal diffusion length is the smallest, and where the phase lag and the heating-detection distance was sufficiently linear.

Therefore, it was possible to accurately measure the thermal diffusivity in the optimum analysis region. The thermal diffusivity of the pure copper sample was then evaluated with actual measurements.

**Figure 2:**
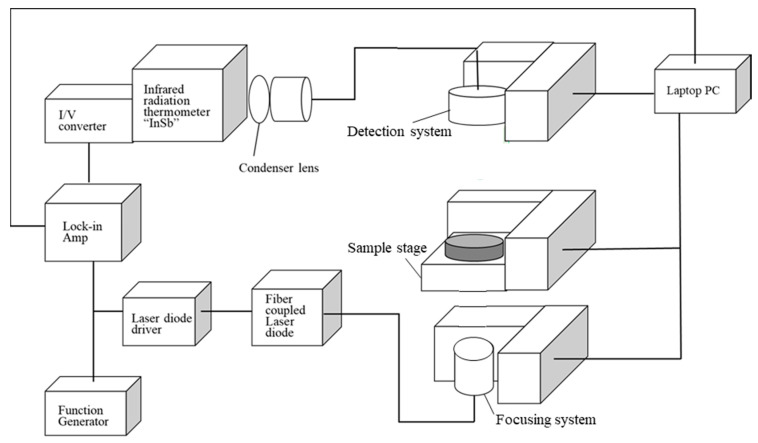
Configuration of thermowave analyzer (TA).

The TA manufactured by Bethel Co., Ltd. (Ibaraki, Japan) was used. Figure 2 shows a block diagram of the device. The signal generated by the function generator causes the laser to be modulated into a sine wave of frequency, *f*. The focusing system, sample stage, and detection system are each controlled by a PC, and it is possible to perform measurements while mechanically moving the measurement position. The temperature response on the back side of the sample is detected by the radiation thermometer, input to the lock-in amplifier, and then the result is the output on the PC.

## 3. Results and Discussion

### 3.1. Evaluation of Gaussian Distribution of Laser

**Figure 3:**
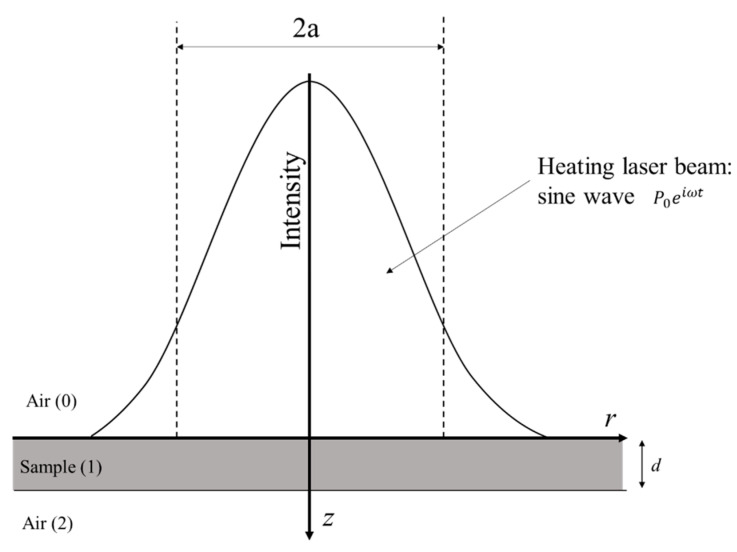
Schematic of the heating laser beam.

The simulation model was assumed as an infinite flat plate with a thickness of *d*. Figure 3 shows the schematic diagram of the heating laser beam. The intensity of the heated laser beam produced by the laser diode was driven directly by a sinusoidal drive current. The intensity of the laser beam with Gaussian distribution due to the heating of the surface of the sample *I* is shown by the following equation.

(4) $$I=\frac{E}{4\pi a^{2}}\exp\left\{-2{\left(r/a\right)}^{2}\right\}\left\{1-\cos\left(2\pi ft\right)\right\}$$

where *E*, the total absorption energy of the laser, is 8.0 × 10^−2^ W, *r* is the distance from the center of the laser, and a is the radius of the laser beam with Gaussian distribution, which was detected by the CMOS camera. The determined radius of the heating laser beam is 0.151 mm.

The temperature response when the sample surface is heated with the intensity-modulated Gaussian beam with angular frequency *ω* is shown by the following equation.

(5) $$T\left(r,z,t\right)=\left[\int_{0}^{\infty }\tau \left(p,z,t\right)pJ_{0}\left(px\right)dp\right]\exp\left(i\omega t\right)+c.c$$

where *τ* is expressed by the Hankel transformation [18] of the surface temperature *T* as follows.

(6) $$\tau \left(p,z,t\right)=\Gamma \left(p\right)\exp\left(-bz\right)+A\left(p\right)\exp\left(-\beta_{1}z\right)+B\left(p\right)\exp\left(\beta_{1}z\right)$$

The parameter of Equation (6) is as follows.

(7) $$\Gamma \left(p\right)=\frac{pb}{2\pi \lambda_{1}}\cdot \frac{\left[-{\left(pa\right)}^{2}/8\right]}{\beta_{1}^{2}-\alpha^{2}}$$

(8) $$\beta_{0}^{2}=p^{2}+i\omega /k_{0}$$

(9) $$\beta_{1}^{2}=\frac{\lambda_{x}}{\lambda_{z}}p^{2}+i\omega /k_{1}$$

(10) $$\beta_{2}^{2}=p^{2}+i\omega /k_{2}$$

(11) $$k_{1}=\frac{\lambda_{z}}{\rho_{1}c_{1}}$$

(12) $$k_{1}=\frac{\lambda_{i}}{\rho_{i}c_{i}}\;\left(i=0,2\right)$$

where $\lambda_{i}$, $\rho_{i}$, $c_{i}\left(i=0,2\right),\;\lambda_{z},\;\rho_{1},\;\mathrm{and}\;c_{1}$ are the thermal conductivity of air, density of air, the specific heat capacity of air, the in-plane thermal conductivity of the sample, density of the sample, and the specific heat capacity of the sample, respectively.

The boundary conditions of Equation (5) are as follows.

(13) $$\lambda_{0}{\frac{\partial T_{0}}{\partial_{z}}|}_{z{}_{=0}}=\lambda_{1}{\frac{\partial T_{1}}{\partial_{z}}|}_{z{}_{=0}}\;\;\;\;\;\;\lambda_{1}{\frac{\partial T_{1}}{\partial_{z}}|}_{z{}_{=d}}=\lambda_{2}{\frac{\partial T_{2}}{\partial_{z}}|}_{z{}_{=d}}$$

(14) $${T_{1}|}_{z{}_{=d}}={T_{2}|}_{z{}_{=d}}\;\;\;\;\;\;{T_{0}|}_{z{}_{=0}}={T_{0}|}_{z{}_{=0}}$$

*A*(*p*) and *B*(*p*) are then represented as follows.

(15) $$A\left(p\right)=\left(1-g\right)\left(\gamma -c\right)\exp\left(-bd\right)-\left(\gamma +g\right)\left(1+c\right)\exp\left(\beta_{1}d\right)\cdot \Gamma \left(p\right)/H\left(p\right)$$

(16) $$B\left(p\right)=\left(1+g\right)\left(\gamma -c\right)\exp\left(-bd\right)-\left(\gamma +g\right)\left(1-c\right)\exp\left(-\beta_{1}d\right)\cdot \Gamma \left(p\right)/H\left(p\right)$$

where b is the absorption coefficient of the sample surface (1.55 × 10^9^)

(17) $$H\left(p\right)=\left(1+g\right)\left(1+c\right)\exp\left(\beta_{1}d\right)-\left(1-g\right)\left(1-c\right)\exp\left(-\beta_{1}d\right)$$

and

(18) $$g=\frac{\lambda_{0}\beta_{0}}{\lambda_{1}\beta_{1}},c=\frac{\lambda_{2}\beta_{2}}{\lambda_{1}\beta_{1}},\gamma =\frac{b}{\beta_{1}}$$

Herein *T*(*r*, *z*, *t*) is referred to as *T*(*r*), and $\lambda_{0},\lambda_{2}$ are negligibly small.

### 3.2. Spatial Sensitivity Distribution of Detector

The sensitivity of the infrared detector as a function of the spatial distribution was measured as follows [15], whereby a distribution of the intensity of the infrared-ray is denoted by *I*(*x*). The measured temperature distribution function U(τ) corresponds to the convolution of *I*(*x*) and the space distribution of the sensitivity *S_x_*(*x*), and can be expressed by the following equation:

(19) $$U\left(\tau \right)=\int_{-\infty }^{\infty }I\left(x\right)S_{x}\left(x-\tau \right)d\tau$$

Assuming that *I*(*x*) is replaced by the step function given by Equation (5), the derivative of U(τ) of Equation (4) provides the space distribution *S_x_*(*x*) as shown in Equation (6).

(20) $$I\left(x\right)=\{\begin{array}{c} m\;\mathrm{for}\;x<0\\ n\;\mathrm{for}\;x\;\geq 0 \end{array}$$

(21) $$\frac{\partial U\left(\tau \right)}{\partial \tau }=\left(m-n\right)S_{x}\left(\tau \right)$$

In addition, the temperature distribution of a sample propagates in the concentric circular at the actual measurement. Therefore, we cannot consider the measured signal *I*(*x*) one-dimensional, thus, we derived the spatial distribution of sensitivity of the axial symmetry against the center of the detector by the spatial distribution of sensitivity *S_x_*(*τ*).

In this study, as sample thickness cannot be ignored, the influence of the thickness of the sample must be evaluated. The experimental procedure to obtain the spatial distribution of the sensitivity of the experimental temperature is as follows. The sample for the spatial distribution measurement of the sensitivity of the infrared detector was used with the plate heater to heat evenly. Half of the surface of the heater sample was uniformly coated with graphite splay (DGF, Nippon Senpaku Kogu KK). Regarding the difference of the radiated infrared-ray between the graphite-coated surface and uncoated surface, the step function *I*(*x*) was obtained through the difference of the intensity of infrared-ray, which was detected by the infrared detector. An electrical current of 5.4 W was applied to the heater sample to maintain a constant temperature, and the intensity of the infrared-ray was measured from the non-coated surface (negative side) to the coated surface (positive side).

**Figure 4:**
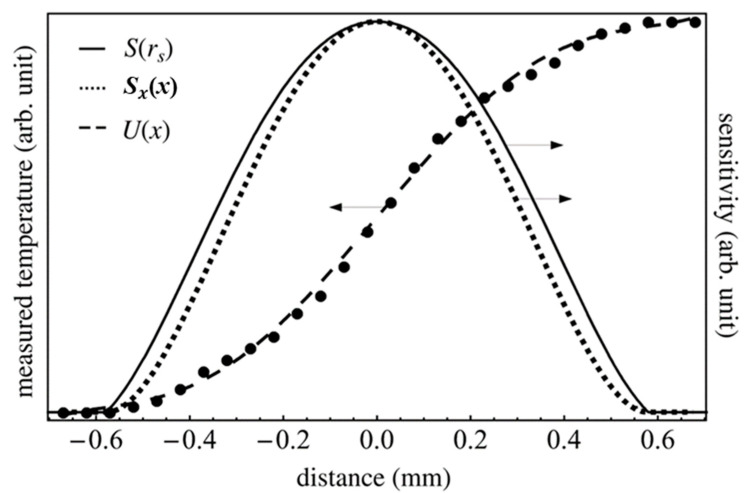
Detected temperature distribution of the plate heater and spatial resolution of the infrared detector. For the negative *r_s_*, the values of *S*(−*r_s_*) were plotted as *S*(*r_s_*) was axisymmetric for *r_s_* = 0.

**Figure 5:**
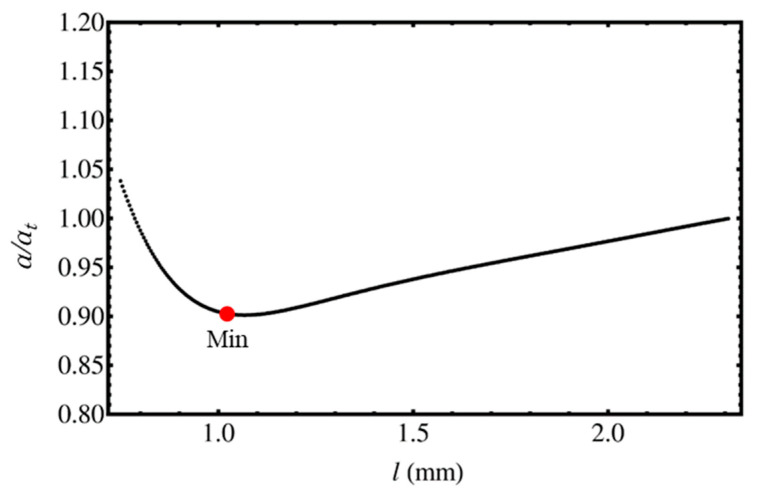
Change in the ratio of thermal diffusivity calculated from the phase lag at a thermal diffusion length with respect to the heating-detection distance.

Figure 4 shows the detected temperature distribution of the heater sample and the spatial resolution of the infrared detector. The dots represent the experimental data of the infrared-ray intensity which was detected by the infrared detector. The measurement data at each point, which is drawn as a closed circle, was 50 μm. The dashed line represents the function of the temperature distribution U(τ). The derivative against *x* of the temperature distribution function U(τ) corresponds to the spatial distribution of sensitivity *S_x_*(*x*). The spatial distribution of sensitivity *S_x_*(*x*) is represented by the dotted line in Figure 5. As the spatial distribution of the sensitivity *S_x_*(*x*) was detected in the inflection point range from −0.6 to 0.6 mm, the distribution of the sensitivity at 0 was determined to be less than −0.6 mm and greater than 0.6 mm. The maximum of *S_x_*(*x*) is located at the boundary of the coated and non-coated surface, which corresponded to *x* = 0 mm. The *S*(*r_s_*) of the distance of the radial direction from the center of the detector *r_s_* can be described using the following equation.

(22) $$S\left(r_{s}\right)=a_{0}+a_{1}r_{s}+a_{2}r_{s}^{2}+a_{3}r_{s}^{3}+a_{4}r_{s}^{4}$$

The maximum of $S\left(r_{s}\right)$ is provided as follows.

(23) $${\frac{\partial S\left(r_{s}\right)}{\partial r_{s}}|}_{r_{s}=0}=0$$

In Equations (22) and (23), $a_{1}$ is 0. Then *S*(*r_s_*) correspond to the value at distance of the spatial distribution of the sensitivity *S_x_*(*x*). The relationship of the spatial distribution of the sensitivities is as follows:

(24) $$S_{x}\left(x\right)=\int_{0}^{\infty }S\left(\sqrt{x^{2}+y^{2}}\right)dy$$

*y* is a component orthogonal to *x*. In the four data points obtained by Equation (24), $a_{0}$, $a_{2}$, $a_{3}$ and $a_{4}\;$ of Equation (22) were determined by solving the simultaneous equations. The solid line in Figure 5 depicts the spatial distribution of the sensitivity *S*(*r_s_*).

### 3.3. Thermal Diffusivity Analysis Method Using Contour Plot

During the evaluation of the thermal diffusivity in the horizontal direction of the sample, the thermal diffusivity was determined from the gradient by plotting the phase lag against the distance. However, the thermal diffusivity evaluated from the gradient of the phase lag was different from the actual thermal diffusivity due to the position of the distance *l*. Thus, the estimation of the thermal diffusivity was performed through the following two steps:

1. The range, where the phase lag shows good linearity with respect to the distance *l*, was determined.
2. A numerical table of the ratio of the true values of thermal diffusivity and the thermal diffusivity which were calculated by Equation (3) using the phase lag obtained by the calculation were prepared.

Using the laser intensity and the spatial sensitivity distribution of the detector obtained in Section 3.1, the temperature response *J*($l_{h}$) from the sample surface is expressed by the following equations from Equations (8) and (24).

(25) $$J\left(l_{h}\right)=\int_{-\infty }^{\infty }\int_{-\infty }^{\infty }T\left(\sqrt{x^{2}+y^{2}}\right)S\left(\sqrt{{\left(l_{h}-x\right)}^{2}+y^{2}}\right)dxdy$$

where $l_{h}$ is the distance of the detection center from the heating center of the temperature measuring surface. The phase lag was calculated by Equation (25) to obtain the calculated thermal diffusivity *α* by Equation (3) at each $l_{h}$. The heat transfer parameter table was obtained from the calculated ratio of the true thermal diffusivity, *α_t_*, to the calculated thermal diffusivity, *α*.

In Figure 5, the minimum point, *Min*., was obtained at each sample thickness and each thermal diffusion length, with the slope of the phase difference, *θ*, being the center of the analytical region where the slope was most stable. At the minimum point, the change in the thermal diffusivity value with the change in *l* is the smallest. The ratio *α_t_*/*α* when deriving can be obtained the minimum point and used as the correction coefficient. Using the collected data, a contour map was created where the analysis distance *l* was uniquely determined by *α*/*f* which is reciprocal of the thermal diffusion length *μ*. The detailed procedure to drive thermal diffusivity is described in following chapter.

**Figure 6:**
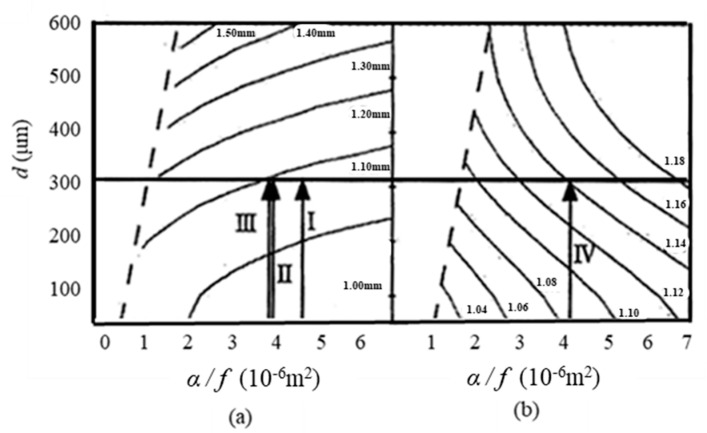
Contour plot for deriving the thermal diffusivity using the optimal heating-detection distance: (**a**) distance for analysis, (**b**) corrective coefficient. I, II, III, and IV are referred to Figure 7.

Figure 6a shows the matrix used to derive the appropriate analysis distance. As the thickness of the measurement sample increased, the appropriate analysis distance also increased. The contour line value refers to the center point of the analysis distance. The intersection point of the contour line with the line subtracted by the thickness of the measurement sample is an appropriate analysis distance. The value of the contour line refers to the the correction coefficient, which is obtained from the intersections as seen in Figure 6a,b shows the diagram of the correction coefficients for obtaining the actual thermal diffusivity from an apparent thermal diffusivity obtained from an appropriate analysis distance. Figure 6b was drawn from the spatial distribution of the detection sensitivity and the Gaussian distribution of the heating laser.

As shown in Figure 6, the maximum value of *x*-axis is approximately 7 × 10^−6^ m^2^ owing to the large thermal diffusion length causing the propagation of the temperature fluctuation to be reflected on the end face of the sample. The range of the *y* axis is from 0.05 to 0.60 mm because it is impossible to obtain a region where the gradient of the phase lag is stable when sample thickness exceeds 0.60 mm. Furthermore, the region drawn without a contour line and delineated by a dotted line is due to the shortness of the thermal diffusion length *μ*, resulting in the small signal strength of the temperature response not satisfying the analysis requirement.

The evaluation of the actual thermal diffusivity using Figure 6 was performed as follows.

1. An arbitrary analysis distance is decided, and the intersection of contour lines of a point obtained by an apparent thermal diffusivity obtained from an arbitrary analysis distance and a line drawn to the thickness of the measurement sample are compared.
2. The analysis distance is set to a more appropriate distance. This operation is carried out until the apparent thermal diffusivity value that can be obtained converges.
3. An actual thermal diffusivity is obtained by using the apparent coefficient of thermal diffusivity obtained by using the correction coefficient given by Figure 6b. The correction factor given is the value of the contour line present at the intersection of the line drawn to the thickness of the measurement sample and the point given by the thermal diffusivity obtained from the appropriate analysis distance.

### 3.4. Evaluation of Thermal Diffusivity for Copper Samples

**Figure 7:**
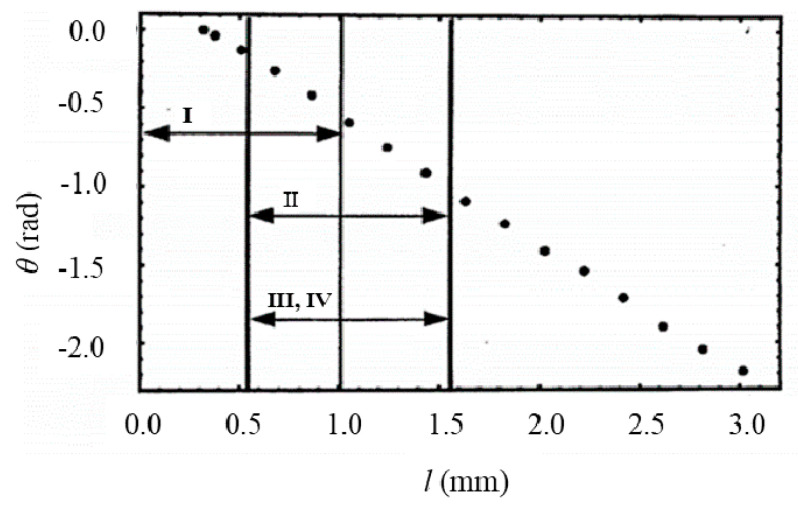
Results of phase lag measurements on copper samples.

As a simple study of thermal diffusivity evaluation, using the heat transfer parameter table, the thermal diffusivity of pure copper as measured by the distance-variation method in frequency was 25 Hz. Figure 7 shows the phase lag *θ* for the copper sample at 25 Hz.

To obtain the thermal diffusivity of pure copper, the matrix (Figure 6) was used in the procedure shown below.

1. As shown by line (I) in Figure 7, the tentative thermal diffusivity *α*’_I_, which was estimated to be 1.23 × 10^−4^ m^2^s^−1^, was derived from the slope of the phase lag using Equation (3) in the range of the tentative analysis distance *l*_I_ = 0–1 mm.
2. In the results from *α*’_I_/*f* and thickness of sample *d* (See line (I) in Figure 6), the approximate analytical distance was obtained to be *l*_II_ = 0.58–1.58 mm.
3. As shown by line (II) in Figure 7, the tentative thermal diffusivity *α*’_II_, which was estimated to be 1.06 × 10^−4^ m^2^s^−1^, was derived from the slope of the phase lag using Equation (3).
4. In the results of *α*’_I_/*f* and thickness of sample *d* (See line (II) in Figure 6), the tentative analytical distance was *l*_II_ = 0.59–1.59 mm.
5. In line (II) and (III) in Figure 7, the phase lag in the range of *l*_III_ was equal to that in the range of *l*_II_. Thus, the evaluated value was converged at this stage.
6. In line (IV) in Figure 6, the correction factor, using the tentative thermal diffusivity *α*’_III_, thickness of sample *d*, heating frecuancy *f*, and applied correction factor to *α*’_III_, was evaluated to be 1.14. In these results, the actual thermal diffusivity of pure copper was 1.18 × 10^−4^ m^2^s^−1^. This value is in good agreement with the literature value (1.17 × 10^−4^ m^2^s^−1^).

Although it was impossible to obtain the accurate thermal diffusivity value when evaluating the thermal diffusivity of the position which was not near the center of pure copper, it was to measure the thermal diffusivity of the disk sample, which was 10 mm in diameter, for the laser flash technique. The accuracy of the novel thermal diffusivity analysis was estimated to be ±3%.

## 4. Conclusions

We developed an accurate thermal diffusivity evaluating method considering that the thermal diffusivity analysis with a spot periodic heating conditions and the sensitivity of the infrared radiation thermometer, in addition to the dimensions of the sample. The conclusions are as follows:

1. We examined the thermal diffusion evaluation method using a parameter table based on heat transfer equations using the concept of optimum distance between heating-point and measurement point.
2. In the results of the thermal diffusivity analysis, the thermal diffusivity of pure copper was 1.18 × 10^−4^ m^2^s^−1^. This value is in good agreement with previous studies.
3. The accuracy of the novel thermal diffusivity analysis was estimated to be ±3%.
